# PsaF Is a Membrane-Localized pH Sensor That Regulates *psaA* Expression in *Yersinia pestis*

**DOI:** 10.1128/JB.00165-21

**Published:** 2021-07-22

**Authors:** Joshua D. Quinn, Eric H. Weening, Virginia L. Miller

**Affiliations:** a Department of Microbiology and Immunology, University of North Carolina, Chapel Hill, North Carolina, USA; b Department of Genetics, University of North Carolina, Chapel Hill, North Carolina, USA; Université de Montréal

**Keywords:** PsaE, PsaF, PsaA, pH 6 antigen, *Y. pestis*, pH sensing, ToxR/S, TcpP/H, DegP, Tsp, regulated intramembrane proteolysis, PsaE, *Yersinia pestis*

## Abstract

The Yersinia pestis pH 6 antigen (PsaA) forms fimbria-like structures and is required for full virulence during bubonic plague. High temperature and low pH regulate PsaA production, and while recent work has uncovered the molecular aspects of temperature control, the mechanisms underlying this unusual regulation by pH are poorly understood. Using defined growth conditions, we recently showed that high levels of PsaE and PsaF (two regulatory proteins required for expression of *psaA*) are present at mildly acidic pH, but these levels are greatly reduced at neutral pH, resulting in low *psaA* expression. In prior work, the use of translational reporters suggested that pH had no impact on translation of *psaE* and *psaF*, but rather affected protein stability of PsaE and/or PsaF. Here, we investigated the pH-dependent posttranslational mechanisms predicted to regulate PsaE and PsaF stability. Using antibodies that recognize the endogenous proteins, we showed that the amount of PsaE and PsaF is defined by a distinct pH threshold. Analysis of histidine residues in the periplasmic domain of PsaF suggested that it functions as a pH sensor and indicated that the presence of PsaF is important for PsaE stability. At neutral pH, when PsaF is absent, PsaE appears to be targeted for proteolytic degradation by regulated intramembrane proteolysis. Together, our work shows that Y. pestis utilizes PsaF as a pH sensor to control *psaA* expression by enhancing the stability of PsaE, an essential *psaA* regulatory protein.

**IMPORTANCE** Yersinia pestis is a bacterial pathogen that causes bubonic plague in humans. As Y. pestis cycles between fleas and mammals, it senses the environment within each host to appropriately control gene expression. PsaA is a protein that forms fimbria-like structures and is required for virulence. High temperature and low pH together stimulate *psaA* transcription by increasing the levels of two essential integral membrane regulators, PsaE and PsaF. Histidine residues in the PsaF periplasmic domain enable it to function as a pH sensor. In the absence of PsaF, PsaE (a DNA-binding protein) appears to be targeted for proteolytic degradation, thus preventing expression of *psaA*. This work offers insight into the mechanisms that bacteria use to sense pH and control virulence gene expression.

## INTRODUCTION

Yersinia pestis is a vector-borne bacterial pathogen that cycles between flea and mammalian hosts to cause bubonic plague, one of three forms of the disease plague (1, 2). Bubonic plague is the most common form of the disease in humans and occurs when bacteria are deposited into the dermal layer of skin during the bite of an infected flea. From the skin, bacteria disseminate through lymphatic vessels to a draining lymph node (3, 4), where they proliferate to high numbers, resulting in enlarged lymph nodes known as “buboes,” a hallmark of bubonic plague. At late stages of disease, bacteria enter the bloodstream and can cause a fatal septicemia. Transcriptome analyses have revealed distinct expression profiles within the flea and mammalian host (5–7), suggesting that Y. pestis has regulatory mechanisms to distinguish between these two environments. While the cues that Y. pestis encounters within each host environment are not well defined, temperature is a key distinguishing signal, as many Y. pestis virulence genes necessary for colonizing mammalian hosts are expressed following an upshift in temperature from 26°C to 37°C (7, 8). One such virulence factor upregulated at 37°C is the “pH 6 antigen” (PsaA). PsaA is produced during mammalian infection and is required for full virulence of Y. pestis in multiple murine models of disease (9–13). Despite this impact, the exact role of PsaA during infection is not known. PsaA forms homopolymeric fimbria-like structures on the bacterial cell surface (14), and there is evidence suggesting that PsaA functions to both promote host cell adherence and inhibit phagocytosis (15, 16). Interestingly, high temperature and low pH are both required to activate *psaA* transcription and PsaA production *in vitro* (9, 10, 17). Despite this unusual regulation, the underlying molecular mechanisms have not been thoroughly investigated.

Two regulatory proteins encoded upstream of *psaA*, PsaE and PsaF, are required for *psaA* transcription (10, 14, 17, 18) and are predicted to play a role in the unusual expression pattern of *psaA* (18, 19). PsaE and PsaF belong to a family of regulatory protein pairs that localize to the inner membrane and function as transcriptional activators (20–27). The ToxR/ToxS and TcpP/TcpH protein pairs in Vibrio cholerae are the most studied members of this family (21–23, 28–33). ToxR and TcpP are integral membrane proteins that directly coactivate expression of *toxT*, which encodes the major V. cholerae virulence regulator ToxT (28, 34–36). Like ToxR and TcpP, PsaE contains an N-terminal cytoplasmic OmpR-like DNA-binding domain, a single transmembrane domain, and a C-terminal periplasmic domain (19), and PsaE is predicted to directly activate *psaA* transcription (37). The periplasmic domain of PsaE constitutes ∼25% of the protein, but the contribution of this domain to the function of PsaE is not known.

The topology of PsaE seems well suited to both sense environmental signals and activate gene expression, yet PsaE alone is not sufficient to activate *psaA* transcription. PsaF is also required and appears to act at least in part by enhancing PsaE stability (18). The PsaF-like proteins TcpH and ToxS inhibit degradation of TcpP and ToxR, respectively, and are thus thought to play indirect roles in *toxT* transcription (29, 31, 38, 39). Like TcpH and ToxS, PsaF contains a single transmembrane domain near the N terminus, with the majority of the protein located in the periplasm. PsaF may also enhance the ability of PsaE to activate *psaA* transcription, as the very low levels of PsaE in the absence of PsaF are unable to activate expression of *psaA* (18).

We previously reported that temperature and pH influence levels of both PsaE and PsaF in Y. pestis via distinct posttranscriptional mechanisms (18). High temperature promotes the production of both PsaE and PsaF; a predicted RNA thermometer located in the 5′ untranscribed region (UTR) of *psaE* modulates *psaE* translation, and *psaF* translation is controlled through an independent mechanism by sequences upstream of *psaF* (18). The levels of PsaE and PsaF detected at high temperature are controlled by environmental pH. High levels of both PsaE and PsaF are present at mildly acidic pH (pH 6.3), but at neutral pH (pH 7.3), PsaE is present at very low levels and PsaF is undetectable (18). Since translation of *psaE* and *psaF* does not appear to be impacted by pH, we predicted that additional pH-dependent posttranslational mechanisms were involved. To further understand how pH regulates transcription of *psaA* in Y. pestis, we set out to define the mechanism(s) by which pH influences the levels of PsaE and PsaF. Thus, we analyzed PsaE and PsaF levels following growth in media buffered to different pH levels. Additionally, we identified residues in the periplasmic domains of PsaE and PsaF that influence protein stability. Our data indicate that the stability of PsaE and PsaF is mutually dependent and that both proteins are sensitive to pH, further supporting the idea that Y. pestis utilizes these integral membrane proteins to control the expression of *psaA* in response to precise environmental cues.

## RESULTS

### A distinct pH threshold controls the levels of PsaE and PsaF.

**FIG 1:**
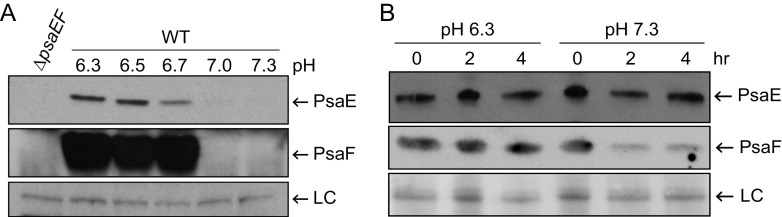
A distinct pH threshold defines PsaE and PsaF levels and impacts PsaF stability. (A) The wild type (WT; YP6) was grown at 37°C in brain heart infusion (BHI) buffered to pH 6.3, 6.5, 6.7, 7.0, and 7.3, and PsaE and PsaF were analyzed via Western blot as described in Materials and Methods. As a control, whole-cell lysates of the Δ*psaEF* mutant (YPA18) grown at 37°C in BHI pH 6.3 were also analyzed. (B) WT was grown in BHI pH 6.3 for 8 h, cells were pelleted, washed and suspended in phosphate-buffered saline (PBS) adjusted to pH 6.3 or pH 7.3 as indicated in Materials and Methods, and PsaE and PsaF were analyzed over time. LC, loading control from Ponceau S-stained membrane.

We previously reported that high levels of PsaE and PsaF are detected at 37°C and pH 6.3 and that both proteins are greatly reduced at 37°C and pH 7.3 (18). Translation of *psaE* and *psaF* is not impacted by pH (18), and thus it is likely that posttranslational mechanisms are involved. To begin to address how PsaE and PsaF are affected by pH, Y. pestis strain CO92 cured of the virulence plasmid pCD1 (YP6; here referred to as the wild type [WT]) was grown at 37°C in brain heart infusion (BHI) buffered to pH 6.3, 6.5, 6.7, 7.0, or 7.3. Consistent with our previous findings (18), high levels of PsaE and PsaF were detected at pH 6.3, and levels were much lower at pH 7.3 (Fig. 1A). While both proteins were readily detected at pH 6.5 and pH 6.7, PsaF was undetectable at a pH of >6.7. PsaE levels were noticeably reduced at pH 6.7 and were very low at pH 7 and 7.3. These data confirm that pH impacts PsaE and PsaF levels and reveal a distinct threshold between pH 6.7 and 7 that defines the levels of these proteins.

Because pH does not appear to affect translation of *psaF* (18), we hypothesized that pH affects the stability of PsaF. To test this, the WT strain was first grown at 37°C pH 6.3 to the late exponential phase to allow production of PsaE and PsaF. Bacterial cells were then pelleted and suspended in phosphate-buffered saline (PBS) (a nutrient-limiting condition) at pH 6.3 or pH 7.3, and PsaF levels were monitored over time (Fig. 1B). At pH 7.3 in PBS, PsaF levels were reduced within 2 h, whereas a similar change in PsaF levels was not observed at pH 6.3 in PBS. A comparable number of bacteria were recovered after 2 h from both conditions, and this was comparable to the number of bacteria present when they were initially suspended in PBS (data not shown), indicating that cell viability did not contribute to the changes in PsaF levels at pH 7.3. In contrast, once PsaE was produced during growth at pH 6.3, its stability was minimally affected by the shift to pH 7.3 in PBS (Fig. 1B). These data suggest that PsaF is less stable at pH 7.3 than at pH 6.3, but PsaE is relatively stable at both pH levels under these conditions. The relative stability of PsaE at pH 7.3 could be explained by low levels of PsaF being sufficient to stabilize PsaE, or it could indicate that, once produced, PsaE is stable under these conditions.

### DegP and Tsp influence levels of PsaE.

As with PsaF, translation of *psaE* is not affected by pH (18), and we thus predicted that during bacterial growth, pH may also control the stability of PsaE. Regulated intramembrane proteolysis (RIP) is a two-step degradation of membrane proteins that occurs in most domains of life, and recent work shows that bacteria utilize RIP to control virulence gene expression (40, 41). During RIP, a target protein is sequentially cleaved, first by a periplasmic protease (site 1 protease) and then by an integral membrane protease (site 2 protease), resulting in complete degradation of the target protein. The PsaE-like proteins, TcpP and ToxR, are degraded via RIP (30, 38, 39), and ToxS and TcpH (PsaF-like proteins) inhibit degradation of ToxR and TcpP, respectively (29, 31). Therefore, we wanted to determine if PsaE also could be subjected to proteolytic degradation via RIP. Tsp and YaeL (RseP) are the site 1 and site 2 proteases, respectively, that degrade TcpP in Vibrio cholerae, and since deletion of *tsp* and/or *yaeL* restores the ability of TcpP to activate transcription of *toxT* (38, 39), we speculated that preventing degradation of PsaE at pH 7.3 would restore transcription of *psaA*. To test this, genes predicted to encode periplasmic (site 1; *tsp*, *degS*, *degQ*, and *degP*) and membrane (site 2; *rseP*, *ypfJ*, *ypo0398*, and *ftsH*) proteases were mutated and *psaA* expression was analyzed after growth at 37°C and pH 6.3 or pH 7.3. The insertion mutations in *degS*, *degQ*, *ypfJ*, *ypo0398*, and *ftsH* did not impact *psaA* expression, suggesting that the proteases encoded by these genes are not required for degradation of PsaE. However, we cannot exclude the possibility that these insertions did not completely disrupt gene function.

**FIG 2:**
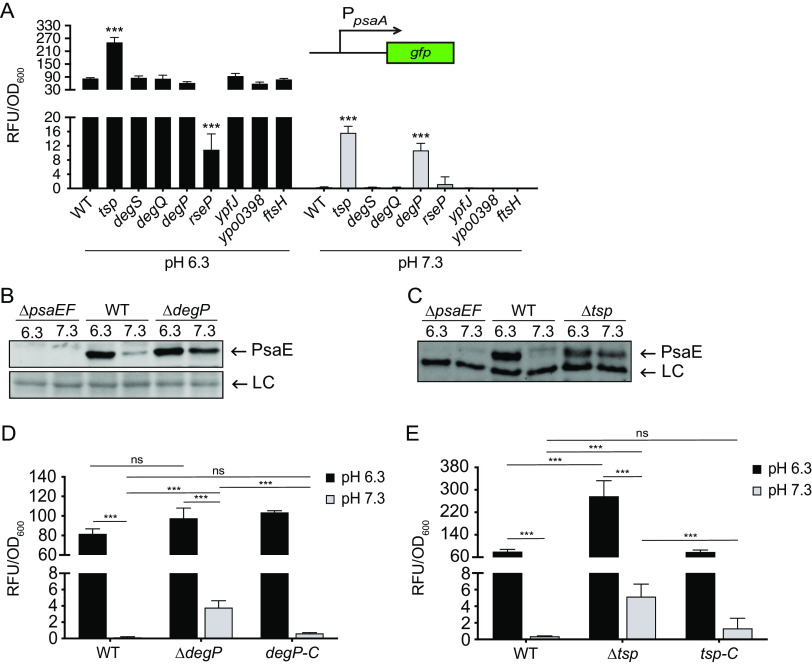
DegP and Tsp contribute to low levels of PsaE and *psaA* transcription at neutral pH. Strains with mutations in putative protease genes were grown at 37°C for 8 h in buffered BHI, and *psaA* transcription and PsaE were analyzed as indicated in Materials and Methods. (A) The *psaA*-*gfp* reporter (pEW102) was introduced into the indicated strains with insertion disruption mutations in the indicated gene, and expression was measured. ***, *P < *0.0001; one-way analysis of variance (ANOVA) and Dunnett’s multiple-comparison test. (B) Whole-cell lysates of WT (YP6), Δ*psaEF* (YPA18), Δ*degP* (YPA425), and Δ*degP* Δ*psaEF* (YPA469) strains grown at 37°C in BHI buffered to pH 6.3 and 7.3 were probed for PsaE via Western blot. LC, loading control. (C) Whole-cell lysates of WT, Δ*psaEF*, Δ*tsp* (YPA350), and Δ*tsp* Δ*psaEF* (YPA379) strains were grown and analyzed as described for panel B. A band that reacted with anti-PsaE serum was used as a loading control (LC). (D) The WT strain, the Δ*degP* strain, and the strain with *deg*P strain restored at the native site (*degP-C*; YPA476) were transformed with *psaA*-*gfp*, and expression was measured. Student’s *t* test was used to compare mean values. ***, *P < *0.0001; ns, not significant. (E) The WT strain, the Δ*tsp* strain, and the strain with *tsp* restored at the native site (*tsp-C*; YPA438) were transformed with *psaA*-*gfp*, and expression was measured. Student’s *t* test was used to compare mean values. ***, *P < *0.0001; ns, not significant.

Of the eight mutants tested, only the *tsp* and *degP* insertion mutants exhibited increased *psaA* expression at pH 7.3 relative to that of the WT strain (Fig. 2A). Mutations in the *tsp* and *degP* genes largely attenuated Y. pestis growth, indicating these proteases play a critical role in Y. pestis physiology. Despite the lower growth rate of these mutants, these data suggest that DegP and Tsp individually contribute to low *psaA* expression under this noninducing condition. The *rseP* insertion mutant had reduced *psaA* expression at pH 6.3 and was severely attenuated for growth on agar plates and in broth medium. We were unable to generate an Δ*rseP* mutant, which is consistent with previous reports that *rseP* is an essential gene in Y. pestis (42, 43). Thus, it is difficult to determine if RseP plays a role in PsaE degradation or if the effect on *psaA* expression of the *rseP* insertion mutant is a consequence of the growth defect.

The increase in *psaA* expression in the *degP* and *tsp* mutants at 37°C and pH 7.3 suggested that PsaE levels and/or activity may be partially restored by loss of these site 1 proteases. As insertion disruption mutations can have polar effects on downstream genes, we constructed individual *degP* and *tsp* deletion mutants (Δ*degP* and Δ*tsp*). These deletion mutants were then tested to determine if the altered *psaA* expression at pH 7.3 was associated with changes in PsaE levels. The WT and mutant strains were grown at pH 6.3 and pH 7.3, and PsaE was analyzed via Western blot. The amount of PsaE present at pH 7.3 was elevated in both the Δ*degP* and Δ*tsp* mutants relative to that in WT, suggesting that increases in *psaA* transcription were due to an increase in PsaE levels (Fig. 2B and C). Yet, PsaF remained undetectable in both mutants at pH 7.3, indicating that Tsp and DegP are not individually responsible for low levels of PsaF under this condition (see Fig. S1 in the supplemental material).

To validate the impact of the predicted site 1 proteases on *psaA* expression, strains with *degP* or *tsp* restored were generated by reintroducing *degP* (YPA476) or *tsp* (YPA438) at the native site of the Δ*degP* and Δ*tsp* mutants, respectively. As seen with the insertion mutants, expression of *psaA* in both the Δ*tsp* and Δ*degP* mutants was higher relative to that in the WT at pH 7.3, suggesting that the absence of these proteases is sufficient to partially restore PsaE activity at pH 7.3 (Fig. 2D and E). Reintroduction of *tsp* and *degP* into the Δ*tsp* and Δ*degP* mutants resulted in *psaA* expression comparable to that of the WT at 37°C pH 7.3, indicating that the observed effect of the Δ*tsp* and Δ*degP* mutations was due to the loss of Tsp and DegP, respectively, rather than due to secondary site mutations. While expression of *psaA* increased in the Δ*degP* mutant (4-fold) and in the Δ*tsp* mutant (5-fold) relative to that in the WT at pH 7.3, expression levels remained low relative to those at pH 6.3. It is possible that this is due to the potential redundancy in Tsp and DegP activity. Our attempt to generate a Δ*degP* Δ*tsp* double mutant to examine this was unsuccessful, likely due to the significant impacts on growth observed with the individual mutants. Under the growth conditions used for these assays, the final optical density at 600 nm (OD_600_) for the *degP* and *tsp* mutants were 17% and 24% that of the WT at pH 7.3, respectively. Together, these data suggest DegP and Tsp individually influence PsaE levels, but it is difficult to determine if these proteases have redundant roles at 37°C and pH 7.3.

### Cysteine residues in PsaE are required for PsaE and PsaF stability.

**FIG 3:**
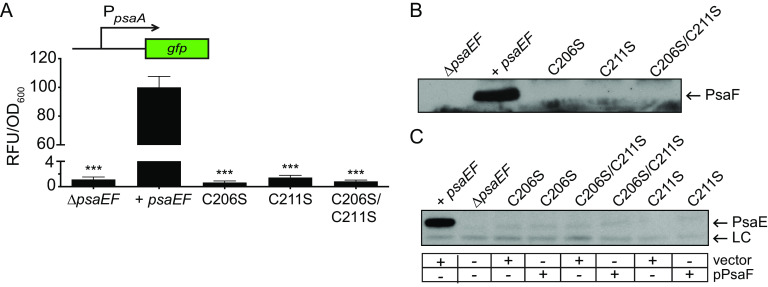
Cysteine residues in the PsaE periplasmic domain impact PsaE and PsaF stability. Strains containing alleles encoding wild-type PsaE or PsaE mutants with cysteine-to-serine substitutions were grown at 37°C in BHI buffered to pH 6.3 or 7.3, and *psaA* transcription, PsaF, and PsaE were analyzed as indicated in Materials and Methods. (A) The *psaA-gfp* reporter was introduced into the Δ*psaEF* mutant (YPA18), or into derivations of the Δ*psaEF* mutant expressing wild-type *psaEF* (YPA260; +*psaEF*), *psaE*_C206S_ (YPA275; C206S), *psaE*_C211S_ (YPA277; C211S), or *psaE*_C206S/C211S_ (YPA276; C206S/C211S), and *psaA* expression was measured. ***, *P < *0.0001 using one-way ANOVA and Dunnett’s multiple-comparison test. (B) Whole-cell lysates from the same strains shown in panel A (lacking *psaA-gfp*) were analyzed for PsaF via Western blot. (C) Whole-cell lysates from the strains shown in panel B transformed with pPsaF or vector (pWKS30) were used to analyze PsaE. A band that cross-reacted with anti-PsaE serum was used as a loading control (LC).

While PsaE is thought to directly activate *psaA* transcription via its DNA-binding domain (37), the role of the periplasmic domain is unknown. Cysteines in the periplasmic domain affect the stability of TcpP and are predicted to impact the function of ToxR (33, 44–47). Like ToxR and TcpP, PsaE contains two cysteine residues in its periplasmic domain. PsaE variants with individual cysteine-to-serine substitutions (PsaE_C206S_ and PsaE_C211S_) and a double cysteine-to-serine substitution (PsaE_C206S/C211S_) were constructed. Expression of *psaA* was similar to that in the Δ*psaEF* mutant in all three PsaE cysteine-to-serine mutants, indicating the importance of these cysteine residues (Fig. 3A).

In V. cholerae, substitution of either cysteine in the TcpP periplasmic domain greatly reduces the stability of both TcpP and TcpH (33). To determine if the cysteine substitutions in PsaE impacted the stability of PsaF, the PsaE cysteine-to-serine mutants were grown at pH 6.3, and PsaF was analyzed via Western blot (Fig. 3B). Strikingly, PsaF was undetectable in all three PsaE mutants. Because the *psaE* open reading frame overlaps with that of *psaF*, it is possible that the nucleotide alterations encoding PsaE_C206S_ (25 nucleotides upstream of *psaF*) or PsaE_C211S_ (10 nucleotides upstream of *psaF*) disrupted translation of *psaF*. As PsaF is required for PsaE stability (18), a disruption of *psaF* translation could lead to an absence of PsaE in strains encoding PsaE cysteine-to-serine mutations. To test this, *psaF* was expressed in *trans* (pPsaF) and introduced in the *psaE* cysteine mutants. Introduction of pPsaF into a Δ*psaF* mutant (*psaE^+^*) restores PsaE levels (18); however, the presence of pPsaF was not sufficient to restore detection of PsaE_C206S_, PsaE_C211S_, or PsaE_C206S/C211S_, indicating that reduced translation of *psaF* in these mutants likely was not responsible for the lack of detectable PsaE (Fig. 3C). Together, these data indicate that both cysteines in the C-terminal periplasmic domain of PsaE impact the stability of both PsaE and PsaF.

### PsaF is a pH sensor.

**FIG 4:**
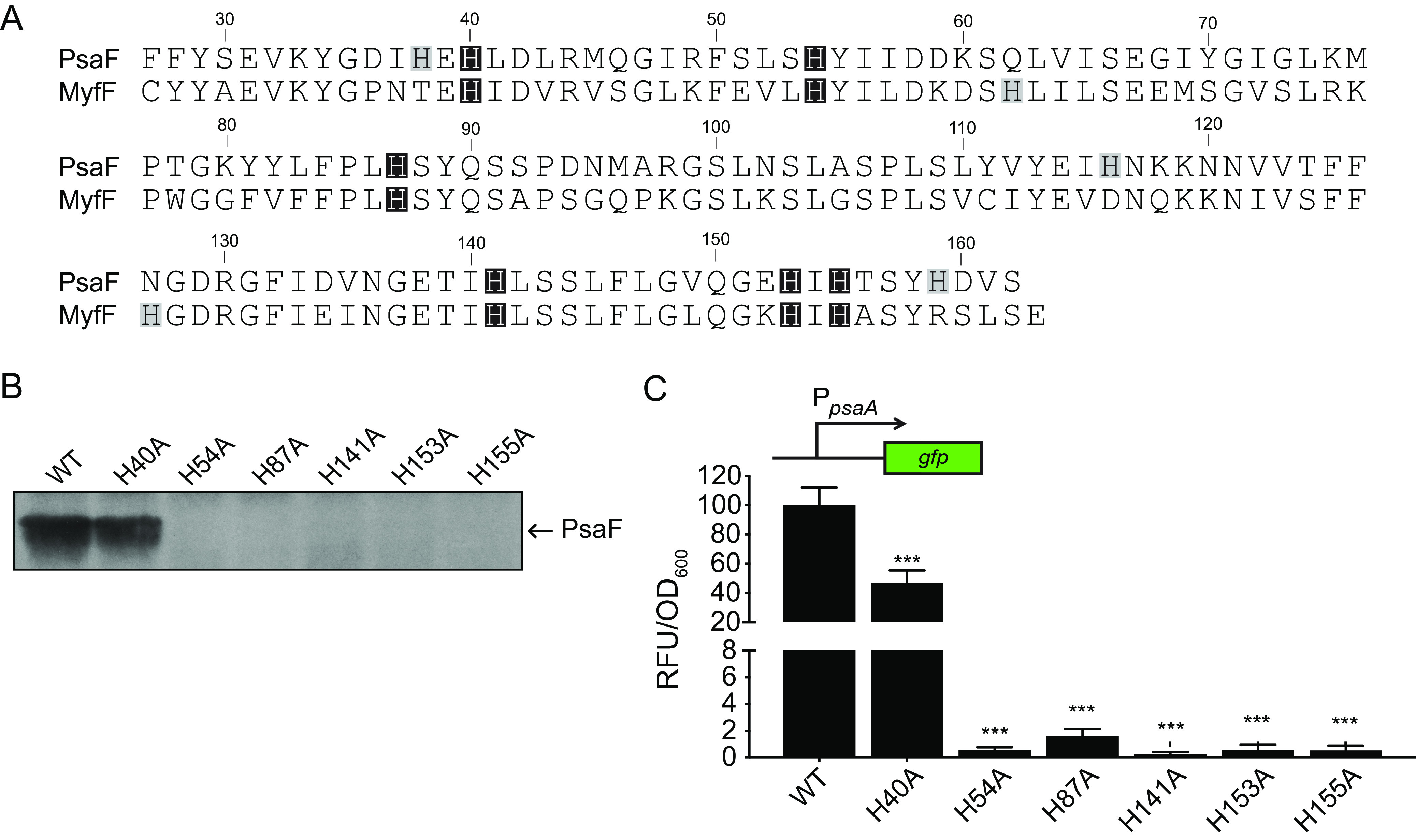
Histidine residues in the PsaF periplasmic domain contribute to PsaF stability and transcription of *psaA*. (A) Amino acid alignment of the PsaF and MyfF periplasmic domains. DNA sequences were obtained from NCBI and translated using Geneious Prime 2019.2.3 (PsaF, Y. pestis CO92, GenBank accession number NC_003143; MyfF, Y. enterocolitica 8081, accession number NC_008800). Histidine residues conserved between both proteins are highlighted with a black box, and histidine residues unique to each protein are highlighted with a gray box. (B) Wild-type and mutant *psaF* alleles were introduced at the native site in the Δ*psaEF* mutant (YPA18) to generate mutants expressing wild-type *psaF* (YPA260; WT), *psaF*_H40A_ (YPA271; H40A), *psaF*_H54A_ (YPA317; H54A), *psaF*_H87A_ (YPA274; H87A), *psaF*_H141A_ (YPA278; H141A), *psaF*_H153A_ (YPA325; H153A), and *psaF*_H155A_ (YPA267; H155A). These strains were grown at 37°C in BHI buffered to pH 6.3, and whole-cell lysates were used to analyze PsaF. (C) These same strains were transformed with *psaA*-*gfp*, grown at 37°C in BHI buffered to pH 6.3, and *psaA* expression was determined. ***, *P < *0.0001 using one-way ANOVA and Dunnett’s multiple-comparison test.

The large periplasmic domain of PsaF seems ideal for sensing environmental cues such as extracellular pH. This region contains nine histidine residues. The pK_a_ of histidine imidazole is ∼6, and histidine protonation at mildly acidic pH levels can impart a “pH-sensing” role shown to modulate the conformation and/or activity of multiple bacterial two-component sensor kinases and viral membrane proteins (48–54). The Yersinia enterocolitica PsaF homologue, MyfF, also contains a histidine-rich periplasmic domain, and six of these histidines are conserved in PsaF (Fig. 4A). While the effect of pH on MyfF has not been reported, the expression of *myfA* (*psaA* homologue) is elevated at acidic pH (55, 56). Thus, we wanted to determine if these histidines drive pH-dependent function and/or stability of PsaF. To test this, *psaF* mutants encoding individual histidine-to-alanine substitutions were expressed with a wild-type *psaE* allele on the chromosome at the native site. Individual substitutions were made for the six histidines shared between PsaF and MyfF. Western blot analysis of lysates from the six individual PsaF mutants grown at 37°C and pH 6.3 indicated that only PsaF_H40A_ was readily detected at levels similar to those of WT PsaF (Fig. 4B). Corresponding with the presence of PsaF, *psaA* was highly expressed only in the WT strain and the mutant PsaF_H40A_ strain (Fig. 4C). A triple His-to-Ala substitution was also made for the three histidines unique to PsaF (PsaF_H38A_, PsaF_H116A_, and PsaF_H159A_). Of note, this triple His-to-Ala substitution mutant also produced PsaA (see Fig. S2 in the supplemental material), suggesting that H40 and the three histidine residues unique to PsaF do not significantly impact PsaF function under this condition. The other five PsaF mutants (H54A, H87A, H141A, H153A, and H155A) were undetectable at pH 6.3, suggesting these residues are essential for PsaF stability under this condition. Consistent with the absence of PsaF, very low levels of *psaA* expression were detected in these mutants (Fig. 4C).

**FIG 5:**
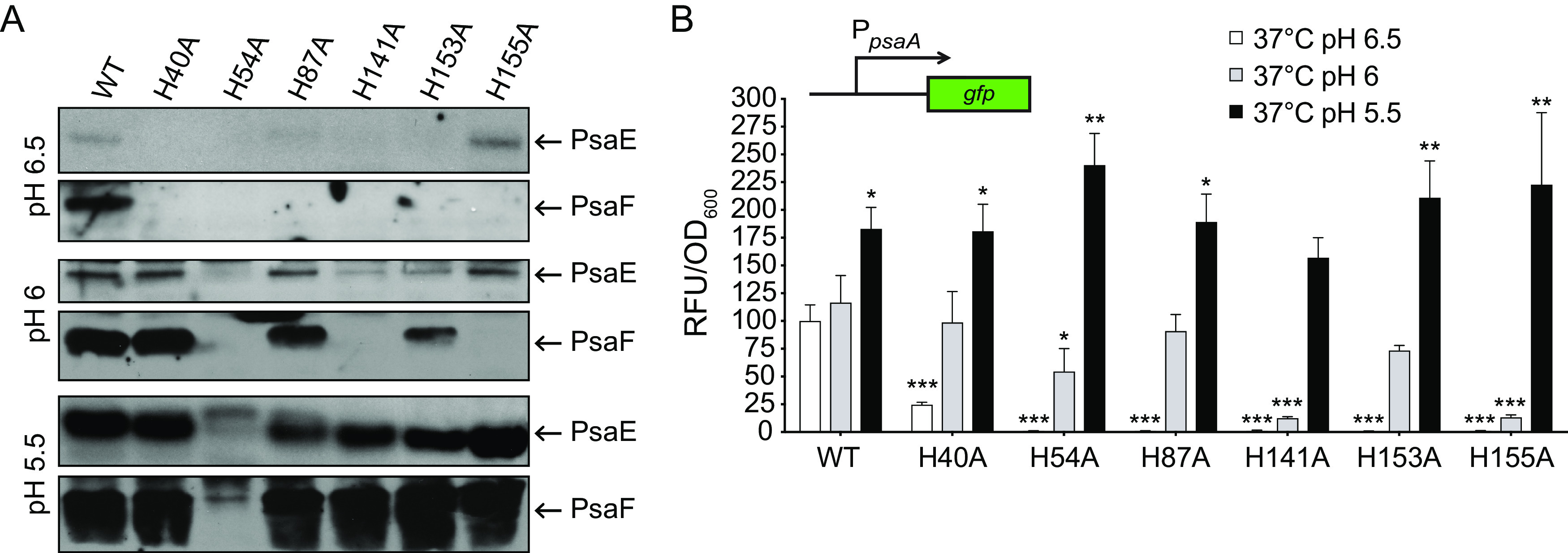
The stability and function of PsaF histidine-to-alanine substitution mutants is restored by low pH. (A) The same strains shown in Fig. 4B were grown at 37°C in BHI buffered to pH 6.5, 6.0, and 5.5, and whole-cell lysates were used to analyze PsaE and PsaF via Western blot. (B) The *psaA*-*gfp* plasmid was introduced into these same strains, grown as in panel A, and expression of *psaA* was determined. ***, *P < *0.0001; **, *P < *0.001; *, *P < *0.01; one-way ANOVA and Dunnett’s multiple-comparison test to compare mean values of each sample to those of the WT at pH 6.5.

Since histidine protonation can influence protein conformation (57), we wanted to determine if the stability of the PsaF histidine substitution mutants was impacted by pH. The same strains described above were grown at 37°C and pH 6.5 or 6.0, and PsaE and PsaF were analyzed by Western blotting. The Y. enterocolitica homologue of *psaA*, *myfA*, is only expressed at an even lower pH, pH 5 (56); therefore, we also grew these strains at 37°C and pH 5.5 for Western blot analysis of PsaE and PsaF. None of the PsaF mutants, including PsaF_H40A_, were detected at pH 6.5, a condition under which WT PsaF is abundant (Fig. 5A). However, in samples from three of the six mutants (PsaF_H40A_, PsaF_H87A_, and PsaF_H153A_), PsaF was detected when the pH was lowered to 6.0. Of these mutant proteins, only PsaF_H40A_ was present at pH 6.3, suggesting that both PsaF_H87A_ and PsaF_H153A_ are not stable at pH of >6.0. When the pH was further acidified to 5.5, all six PsaF mutant proteins were detected (PsaF_H54A_ was detected at a low level), indicating that the stability of PsaF histidine mutants can be rescued by decreasing the pH.

The presence of near-WT levels of PsaE correlated with the presence of each PsaF histidine mutant. One exception to this was that at pH 6.0 or 6.5, PsaE appeared to be present at levels comparable to those in the WT in the absence of PsaF_H155A_. To determine if these PsaF mutant proteins influence *psaA* expression in response to pH, the WT strain and these *psaF* histidine mutants were then assayed for *psaA* expression after growth at 37°C in BHI at pH 6.5, 6.0, and 5.5 (Fig. 5B). Expression of *psaA* in the WT increased when the pH was more acidic, and this corresponded with an apparent increase in levels of PsaE and PsaF (Fig. 5). Expression of *psaA* in the PsaF His-to-Ala mutants was roughly comparable to that in the WT when the stability of PsaF was restored by growth at lower pH. Collectively, these data suggest that these histidine residues of PsaF are involved in sensing pH and impact stability of both PsaF and PsaE.

## DISCUSSION

**FIG 6:**
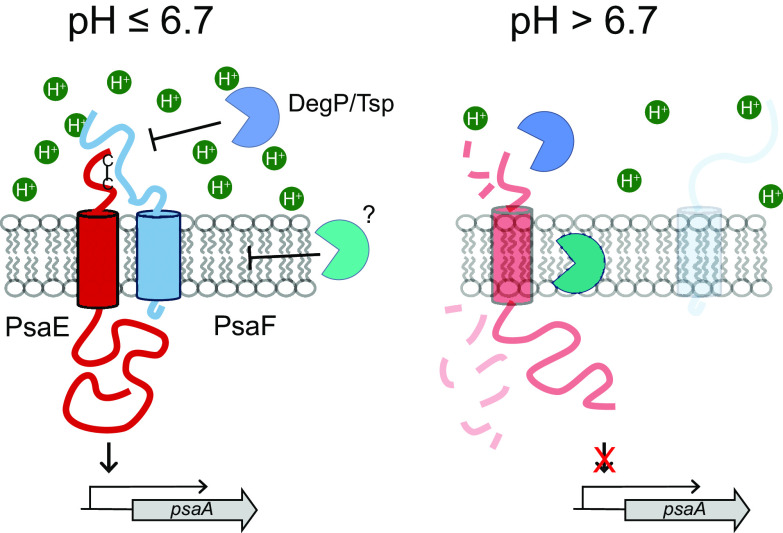
Proposed model for pH control of *psaA* expression by PsaE and PsaF. PsaF contains a histidine-rich periplasmic domain that impacts the folding and stability of PsaF in response to environmental pH. Intrachain disulfide bond formation in the periplasmic domain of PsaE promotes interaction with PsaF at pH of <6.7, thus blocking proteolytic degradation of PsaE via regulated intramembrane proteolysis (RIP). At pH of >6.7, conformational changes in PsaF prevent effective interaction with PsaE, and PsaE is targeted for proteolytic degradation. Expression of *psaA* increases as the pH decreases, and thus pH may also impact the activity of PsaE and PsaF through additional mechanisms that have yet to be determined.

PsaA is a bubonic plague virulence factor and is produced when Y. pestis is grown *in vitro* under high temperature and low pH conditions (9, 10, 17–19). Despite the strong impact of temperature and pH on *psaA* expression, the underlying mechanisms have remained largely unknown. Using defined growth conditions, we recently demonstrated that high temperature and low pH activate *psaA* transcription in Y. pestis, and our work suggests that posttranscriptional regulation of two key *psaA* regulatory proteins, PsaE and PsaF, is primarily responsible for this unusual expression pattern (18). From our previous work, we predicted that posttranslational mechanisms also control the observed pH-dependent effect on PsaE and PsaF levels. Our earlier study examining the absence of PsaE or PsaF in Δ*psaF* or Δ*psaE* mutants, respectively, indicated that PsaE and PsaF exhibit codependent stability (18). Here, we provide evidence that residues in the periplasmic domains of each protein impact their stability. Building upon our previous work, we propose a model by which PsaF uses its histidine-rich periplasmic domain to sense pH (Fig. 6). As PsaE is thought to directly activate transcription of *psaA*, we predict that Y. pestis utilizes PsaF to stabilize PsaE and control *psaA* transcription in response to pH.

Acidic pH is known to influence gene expression in multiple bacteria (58–62). Yet, knowledge of the proteins and mechanisms underlying these pH-sensing pathways is limited. The data presented in this study into how PsaE and PsaF control expression of *psaA* offer insight into the mechanisms bacteria use to sense pH and activate gene expression. We show that a distinct pH threshold (pH > 6.7) determines whether or not PsaF is present. Because translation of *psaF* occurs at both pH 6.3 and pH 7.3 (18), it appears that pH controls protein stability rather than production. After production at pH 6.3, PsaF levels decrease following a shift to neutral pH, whereas levels remain high when left at mildly acidic pH; thus, PsaF functions as a membrane-localized pH-sensor, with its stability linked to extracellular pH. As with other proteins that have been implicated as pH sensors (48–50, 54, 63), PsaF has a periplasmic domain that is ideally positioned for such environmental sensing. This domain contains nine histidine residues, and we found that five were essential for PsaF stability at pH 6.3. The stability of PsaF in these five mutants is rescued by further reducing the pH, indicating that these mutants can sense and respond to this signal. While additional studies are needed to determine how histidines enhance PsaF stability, the involvement of pH suggests that their ionization state plays a role such that their protonation promotes stability. Stability of individual PsaF histidine mutants is rescued by growth at lower pH. One possible explanation for this is that individual residues become protonated within the folded protein at different pH levels. While the pK_a_ of histidine imidazole is ∼6.0, protein structure can influence the pK_a_ of individual residues within a protein (64, 65).

We previously reported that the expression of *psaF* from a plasmid restores PsaE to high levels in a Δ*psaF* mutant, suggesting that one key role of PsaF is to enhance PsaE stability (18). The ability of PsaF His-to-Ala mutants to rescue PsaE stability and activate *psaA* transcription at lower pH levels, when the stability of these PsaF mutants themselves is restored, further supports this role. We predict that low pH alters the structural conformation of PsaF and that this affects its ability to interact with and enhance the stability of PsaE. PsaE is present at very low levels at pH of >6.7, and the absence of PsaF above this pH threshold is likely responsible for the observed loss of PsaE. However, we cannot exclude the possibility that PsaE also senses pH.

Recent work on PsaE-like proteins (ToxR and TcpP) identified proteases involved in targeted protein degradation that indicated these proteins are degraded by RIP (30, 31, 38, 39). Here, we present evidence that DegP and Tsp, two proteases that contribute to ToxR and TcpP degradation, respectively (30, 39, 66), also contribute to the low levels of PsaE observed at pH 7.3. Tsp and DegP are both periplasmic proteases and may directly recognize and target the PsaE periplasmic domain. As PsaF is absent at pH 7.3, it seems likely that PsaF serves to block access of these proteases to PsaE and thus plays a similar role to that of ToxS and TcpH, which inhibit RIP of ToxR and TcpP, respectively (29, 31).

The cytoplasmic N-terminal domain of PsaE contains an OmpR-like winged helix-turn-helix DNA-binding domain that is thought to bind the *psaA* promoter to directly activate *psaA* expression (19, 37). While PsaE-like proteins possess a conserved topology, the primary sequences of their periplasmic domains do not resemble each other, and a full understanding of the function of this domain is lacking. As was observed with TcpP (33), PsaE contains two cysteines in the periplasmic domain and our data indicate that both influence protein stability. Conversely, loss of either cysteine does not as readily impact the overall stability of ToxR but may alter its activity (44–47, 66). In both TcpP and ToxR, the cysteines influence protein conformation (33, 44–46, 66, 67). Notably, mutation of the cysteines in PsaE also negatively impacts PsaF stability, supporting our previous work indicating that the stability of PsaE and PsaF is mutually dependent (18). A similar effect of cysteine mutations in TcpP on TcpH stability has been observed (33). A recent study using the purified periplasmic domains of ToxR and ToxS suggested that intradomain disulfide bond formation of ToxR promotes binding to ToxS (68). This finding raises the possibility that intradomain disulfide bond formation in the periplasmic domain of PsaE is necessary for interaction with PsaF. In this scenario, if PsaF does not fold properly due to pH levels above 6.7 and cannot bind PsaE, then PsaE is susceptible to proteolysis via Tsp and DegP. When unable to interact with PsaE, PsaF itself appears to be unstable. Unlike that of PsaE, loss of Tsp or DegP alone is not sufficient to restore stability of PsaF at pH 7.3. These data are consistent with the observation that cysteine-to-serine mutations of PsaE result in loss of stability of both PsaE and PsaF.

While the topology of PsaE seems to be ideal for both environmental sensing and gene activation, it alone cannot activate *psaA* transcription. In a previous study, the absence of PsaF was associated with low *psaA* expression even when PsaE was produced at low temperatures (18). At pH levels of >6.7, where only low levels of PsaE and no PsaF are detected, there is negligible *psaA* transcription and PsaA cannot be detected. In most cases, PsaE levels were significantly reduced when PsaF was undetectable. The analysis of the PsaF histidine mutants, and their rescue by lowering the pH, indicated that in all growth conditions when both PsaE and PsaF were detectable, *psaA* expression was comparable to that in the WT. Together, these data indicate that PsaF may serve to do more than solely inhibit PsaE degradation and may also influence PsaE activity.

Our data resemble that of a recent report that suggested a direct interaction between the ToxR and ToxS periplasmic domains; the strength of this interaction was negatively impacted at alkaline pH, a growth condition under which ToxR is degraded (32). It is tempting to speculate that an analogous phenomenon occurs with PsaE and PsaF. In a similar manner, pH influences the stability of PsaE. The amount of PsaE is slightly lower at pH 6.7 relative to that at pH 6.3, yet PsaF levels are not altered at this pH range. This may indicate that PsaF and PsaE do not interact as effectively at intermediate pH (e.g., pH 6.7) resulting in reduced amounts of PsaE. In the absence of PsaF at pH of >6.7, levels of PsaE are even further reduced.

Bacteria control protein stability as a means of rapidly modulating gene expression in response to changes in environmental cues via RIP (69), and our data suggest that regulation of PsaE and PsaF stability drives pH-dependent *psaA* transcription. PsaE and PsaF belong to an unusual family of paired transcriptional regulators that are localized to the inner membrane (19, 21, 23), and most studies have focused on the function and regulation of the “PsaE-like” effector (i.e., ToxR and TcpP), likely because of their predicted role as a direct transcriptional regulator. PsaF clearly plays a vital role in enhancing PsaE stability (18), as has been proposed for the PsaF-like proteins TcpH and ToxS (29, 31). Yet despite this role, little attention has been focused on PsaF-like proteins. This work provides new information on the role of PsaF by defining it as the pH sensor contributing to the pH dependent expression of *psaA*. As with PsaE and PsaF, TcpP and TcpH exhibit codependent stability; however, it is unknown if TcpP or TcpH levels are regulated in response to specific environmental signals. It is also not known if the PsaF-like proteins TcpH and ToxS directly function as sensors of specific environmental signals. Our work supports the current paradigm while offering new insights into mechanisms that influence the codependent stability of these protein pairs by revealing critical regulation of PsaF in response to an environmental signal, pH. The observation that PsaE is stable following a shift from pH 6.3 to 7.3 but that PsaF is not further suggests that PsaF serves as the direct pH sensor. Thus, we propose the temperature- and pH-dependent regulation of *psaA* transcription can be explained by temperature-dependent translation of *psaE* and *psaF*, an effect of pH on folding of PsaF and its interaction with PsaE, which in turn impacts the susceptibility of PsaE to proteolysis.

## MATERIALS AND METHODS

### Bacterial strains and growth conditions.

Bacterial strains and plasmids used in this study are listed in Table 1. Y. pestis strains were cultivated on brain heart infusion (BHI) agar (BD Biosciences, Bedford, MA) at 26°C for 48 h and in BHI broth with aeration at 26°C or at 37°C. Escherichia coli strains were grown in LB (BD Biosciences) at 37°C. When indicated, bacteria were grown in BHI broth that was buffered to the indicated pH and filter sterilized as described previously (18). When necessary, antibiotics were added at the following concentrations: kanamycin (Kan), 50 μg/ml; carbenicillin (Carb), 100 μg/ml; and Irgasan (Irg), 2 μg/ml.

**TABLE 1:** Bacterial strains and plasmids

Species, strain, or plasmid	Description^*a*^	Reference or source
Escherichia coli		
DH5α	F^−^ ϕ80Δ*lacZ*M15 Δ(*lac*ZYA-*argF*)U169 *deoP recA1 endA1 hsdR17*(r_K_^−^ m_K_^−^)	Invitrogen
S17-1λpir	Tp^r^ Str^r^ *recA thi pro hsdR hsdM*^+^ RP4::2-Tc::Mu::Km Tn*7* λpir	72
Yersinia pestis		
YP6	CO92, pCD1^−^	11
YPA18	YP6 Δ*psaEF*	18
YPA260	YP18 with pEW104 at the native site	18
YPA425	YP6 Δ*degP*	This work
YPA469	YPA18 Δ*degP*	This work
YPA476	YPA425 with pJQ054 at the native site	This work
YPA350	YP6 Δ*tsp*	This work
YPA379	YPA18 Δ*tsp*	This work
YPA438	YPA350 with pJQ050 at the native site	This work
YPA370	YP6 *tsp*::pJQ014	This work
YPA391	YP6 *degS*::pJQ032	This work
YPA387	YP6 *degQ*::pDF001	This work
YPA395	YP6 *degP*::pDF004	This work
YPA382	YP6 *rseP*::pJQ031	This work
YPA393	YP6 *ypfJ*::pDF002	This work
YPA388	YP6 *ypo0398*::pDF003	This work
YPA396	YP6 *ftsH*::pDF005	This work
YPA275	YPA18 with pEW107 at the native site	This work
YPA277	YPA18 with pEW108 at the native site	This work
YPA276	YPA18 with pEW109 at the native site	This work
YPA273	YPA18 with pEW110 at the native site	This work
YPA271	YPA18 with pEW111 at the native site	This work
YPA317	YPA18 with pEW112 at the native site	This work
YPA274	YPA18 with pEW113 at the native site	This work
YPA278	YPA18 with pEW114 at the native site	This work
YPA325	YPA18 with pEW115 at the native site	This work
YPA267	YPA18 with pEW116 at the native site	This work
Plasmids		
pSR47S	Kan^r^, MobRP4 *ori*R6K *sacB* suicide vector	70
pPROBE-AT	Ap^r^, *gfp* reporter vector	73
pWKS30	Ap^r^ cloning vector	74
pEW102	*psaA* promoter in pPROBE-AT	18
pEW104	*psaEF* and flanking sequences in pSR47S	18
pEW105	*psaEF* promoter and *psaE* in pSR47S	18
pEW106	*psaEF* promoter and *psaF* in pSR47S	18
pPsaF	*psaEF* promoter and *psaF* coding sequence in pWKS30	18
pJQ014	pSR47S with an internal fragment from YP6_1705 (*tsp*)	This work
pJQ032	pSR47S with an internal fragment from YP6_ 3568 (*degS*)	This work
pDF001	pSR47S with an internal fragment from YP6_ 3566 (*degQ*)	This work
pDF004	pSR47S with an internal fragment from YP6_ 3382 (*degP*)	This work
pJQ031	pSR47S with an internal fragment from YP6_ 1051 (*rseP*)	This work
pDF002	pSR47S with an internal fragment from YP6_ 3058 (*ypfJ*)	This work
pDF003	pSR47S with an internal fragment from YP6_ 0398 (peptidase family M48)	This work
pDF005	pSR47S with an internal fragment from YP6_ 3502 (*ftsH*)	This work
pJQ018	*tsp* flanking sequences in pSR47S	This work
pJQ047	*degP* flanking sequences in pSR47S	This work
pJQ054	*degP* coding and flanking sequences in pSR47S	This work
pJQ050	*tsp* coding and flanking sequences in pSR47S	This work
pEW107	*psaEF* and flanking sequences with *psaE*_C206S_ mutant allele in pSR47S	This work
pEW108	*psaEF* and flanking sequences with *psaE*_C211S_ mutant allele in pSR47S	This work
pEW109	*psaEF* and flanking sequences with *psaE*_C206S/C211S_ mutant allele in pSR47S	This work
pEW110	*psaEF* and flanking sequences with *psaF*_H38A/H116A/H159A_ mutant allele in pSR47S	This work
pEW111	*psaEF* and flanking sequences with *psaF*_H40A_ mutant allele in pSR47S	This work
pEW112	*psaEF* and flanking sequences with *psaF*_H54A_ mutant allele in pSR47S	This work
pEW113	*psaEF* and flanking sequences with *psaF*_H87A_ mutant allele in pSR47S	This work
pEW114	*psaEF* and flanking sequences with *psaF*_H141A_ mutant allele in pSR47S	This work
pEW115	*psaEF* and flanking sequences with *psaF*_H153A_ mutant allele in pSR47S	This work
pEW116	*psaEF* and flanking sequences with *psaF*_H155A_ mutant allele in pSR47S	This work

a Str, streptomycin; Kan, kanamycin; Ap, ampicillin; ^r^, resistance; Tp, trimethoprim.

### Plasmid and strain construction.

Primers used in this study are listed in Table 2. All in-frame deletion mutants and mutants in which alleles were introduced at the native site were constructed via allelic exchange using the pSR47S suicide vector (70) as previously described (18). All plasmids were constructed via Gibson assembly (NEB) and were confirmed by sequencing.

**TABLE 2:** Primers used in this study

Primer	Sequence^*a*^ (5′ to 3′)	Description^*b*^
*psaEF*compF	ATCGATCCTCTAGA**GTCGAC**ATTAACGGGGGCGCTGTCTATGG	F pEW104 5′
*psaEF*compR	GCTCTAGAACTAGT**GGATCC**ATAACTCAGTCGCAGACCTATAG	R pEW104 3′
*psaF*_3HA_R	CATTCTTAAATCAAGATGCTCAGCGATATCGCCATATTTCACTTC	R pEW110 internal 3′
*psaF*_3HA_F	GAAGTGAAATATGGCGATATCGCTGAGCATCTTGATTTAAGAATG	F pEW110 internal 5′
*psaF*_H40A_R	CTTAAATCAAGAGCCTCATGGATATCGCCATATTTC	R pEW111 3′
*psaF*_H40A_F	GGCGATATCCATGAGGCTCTTGATTTAAGAATGCAAGG	F pEW111 5′
*psaF*_H54A_R	CAATAATATAAGCTGAGAGGCTAAATCTTATCCC	R pEW112 3′
*psaF*_H54A_F	GATTTAGCCTCTCAGCTTATATTATTGATGATAAGTCT	F pEW112 5′
*psaF*_H87A_R	GACTGATATGAAGCAAGAGGGAATAGGTAATACTTCC	R pEW113 3′
*psaF*_H87A_F	CCTATTCCCTCTTGCTTCATATCAGTCATCCCCTGAT	F pEW113 5′
*psaF*_H141A_R	GAGAAGATAAGGCGATCGTTTCTCCATTGACATC	R pEW114 3′
*psaF*_H141A_F	GGAGAAACGATCGCCTTATCTTCTCTGTTTCTCGGG	F pEW114 5′
*psaF*_H153A_R	CGTATGGATAGCTTCTCCTTGTACCCCGAGAAAC	R pEW115 3′
*psaF*_H153A_F	GGTACAAGGAGAAGCTATCCATACGTCCTATCATG	F pEW115 5′
*psaF*_H155A_R	CATGATAGGACGTAGCGATATGTTCTCCTTGTACCCC	R pEW116 3′
*psaF*_H155A_F	GGAGAACATATCGCTACGTCCTATCATGACGTTAG	F pEW116 5′
JQ076	AAAAAGGATCGATCCTCTAGAGCGCGGATAAAAAGTATTCGCTGG	F pJQ014 5′
JQ077	GCCGCTCTAGAACTAGTGGATCCCGCTATCGTTTTACTCTTCGC	R pJQ014 3′
JQ107	AAAGGATCGATCCTCTAGAGGCCAGCAGCTCCCTAGCCTGG	F pDF004 5′
JQ108	GCTCCACCGCGGTGGCGGCCGCGCCGCCGGAATTACCACGGT	R pDF004 3′
JQ110	AAAAAGGATCGATCCTCTAGAGCCCAGCGAATCGAATGGCCG	F pDF005 5′
JQ111	GCTCCACCGCGGTGGCGGCCGGACACGGGATGCACCGACAC	R pDF005 3′
VM1	GC**GTCGAC**GATGAGCGTGTAGAGGCTGTTGCGCC	F pJQ031 5′
VM2	ATAAGAAT**GCGGCCGC**CCCAACATGCTTACCGTCAACCGC	R pJQ031 3′
VM3	GC**GTCGAC**CAGTACGTCGTGCCGCACCGGCGG	F pJQ032 5′
VM4	ATAAGAAT**GCGGCCGC**CCGATGCCTTCCGGTGTTTCGCC	R pJQ032 3′
VM5	GC**GTCGAC**GAGAGCAGCCGGCCATTCGAAGGC	F pDF001 5′
VM6	ATAAGAAT**GCGGCCGC**GGTTTGATCCCGGCTTTCGCTGCCG	R pDF001 3′
VM7	GC**GTCGAC**GGTCGCTATCCTGATTATTGTGTTGG	F pDF002 5′
VM8	ATAAGAAT**GCGGCCGC**CCGATAAGCGATTACCTTCCGCCTGAG	R pDF002 3′
VM9	GC**GTCGAC**CCACTGAACCTGACATTTCCCGATGG	F pDF003 5′
VM10	ATAAGAAT**GCGGCCGC**GTCATCCACATAAAGGTCAAGGCGAC	R pDF003 3′
JQ080	AAAAAGGATCGATCCTCTAGAGGGTCAAAGTCGGTGCTGAGC	F pJQ050 5′
JQ083	GCCGCTCTAGAACTAGTGGATCCCGCCAGGTTGGTGAGAAGG	R pJQ050 3′
JQ081	GCGACAGGGCGAGATGCAAGCCTAGGTTGGCCTCCGTATC	R pJQ018 up 3′
JQ082	TTGCATCTCGCCCTGTCGCTGCTGCTACCGCTGGGGCAACGG	F pJQ018 down 5′
JQ134	AAAAAGGATCGATCCTCTAGAGGGGCTGCTTGATATTTATAGC	F pJQ054 5′
JQ137	CCGCTCTAGAACTAGTGGATCGGGCACAGGCTGTCAGGCAAGC	R pJQ054 3′
JQ135	ACTGTATCAATACCTTACTGCATATTCGTGTTCTCACTATAC	R pJQ047 up 3′
JQ136	GCAGTAAGGTATTGATACAGTAAGGTATTGATACAGTAAGG	F pJQ047 down 5′
*psaE*C206S_F	AAACAGCAATGAAAGCAAAATCACTTACTCTCATA	F pEW107 internal 5′
*psaE*C206S_R	CTTTCATTGCTGTTTGCATTCCGATTGATCAGAGATAATGACTAAACCAAAATC	R pEW107 internal 3′
*psaE*C211S_F	AAACAGCAATGAAAGCAAAATCACTTACTCTCATA	F pEW108 internal 5′
*psaE*C211S_R	CTTTCATTGCTGTTTGGATTCCGATTGATCACAGATAATGACTAAACCAAAATC	R pEW108 internal 3′
*psaE*C206-211S_F	AAACAGCAATGAAAGCAAAATCACTTACTCTCATA	F pEW109 internal 5′
*psaE*C206-211S_R	CTTTCATTGCTGTTTGGATTCCGATTGATCAGAGATAATGACTAAACCAAAATC	R pEW109 internal 3′

a Restriction sites are shown in bold. Sequence overlap for Gibson assembly is underlined.

b F, forward primer; R, reverse primer.

### (i) In-frame deletions.

The plasmids for in-frame deletions of *degP* and *tsp* were constructed by amplifying ∼500-bp DNA fragments upstream and downstream of the target gene. These fragments were cloned into pSR47S, and the resulting plasmids, pJQ047 (Δ*degP*) and pJQ018 (Δ*tsp*), were introduced into YP6 (WT) or YPA18 (Δ*psaEF*) via conjugation as previously described (18). Briefly, transconjugants were selected on BHI plates with Kan_50_ and Irg_2_. The second recombination event was selected for by streaking Kan^r^ (Kan-resistant)/Irg^r^ colonies onto BHI agar plates containing 5% sucrose. Deleted genes were confirmed by PCR.

### (ii) Reintroduction of *degP* and *tsp* in Δ*degP* and Δ*tsp* mutants.

The plasmids used to reintroduce *degP* and *tsp* at the native site were constructed as follows. The target gene coding sequence, including 500-bp upstream and downstream flanking regions, was amplified and cloned into pSR47S, and the resulting plasmids, pJQ054 and pJQ050, were introduced into YPA425 (Δ*degP*) and YPA350 (Δ*tsp*), respectively, via conjugation and integration of the plasmids, and were identified by selection on BHI plates with Kan_50_ and Irg_2_.

### (iii) Protease insertion mutants.

All plasmids for generating insertion mutations in putative protease genes were constructed by amplifying ∼500-bp DNA fragments of internal coding sequence of the gene. These fragments were cloned into pSR47S, the resulting plasmids (pJQ014, pJQ031, pJQ032, pDF001, pDF002, pDF003, pDF004, and pDF005) were introduced into YP6 via conjugation, and insertion disruption mutants generated by single crossover were identified by selection on BHI plates with Kan_50_ and Irg_2_. The resulting strains are YPA370 (*tsp*), YPA382 (*rseP*), YPA391 (*degS*), YPA387 (*degQ*), YPA393 (*ypfJ*), YPA388 (*ypo0398*), YPA395 (*degP*), and YPA396 (*ftsH*), respectively.

### (iv) *psaE* and *psaF* substitution mutants.

The plasmids for generating *psaE* mutant alleles encoding cysteine-to-serine substitutions were constructed by amplifying the *psaEF* coding sequence with primers containing the substituted nucleotides. These products were then cloned into pSR47S to generate pEW107 (*psaE*_C206S_), pEW108 (*psaE*_C211S_), and pEW109 (*psaE*_C206S/C211S_). The plasmids for generating *psaF* mutant alleles encoding histidine-to-alanine substitutions were constructed using a similar procedure as for the *psaE* cysteine-to-serine mutants, using primers containing the substituted nucleotides. These products were then cloned into pSR47S to generate pEW110 (*psaF*_H38A/H116A/H159A_), pEW111 (*psaE*_H40A_), pEW112 (*psaE*_H54A_), pEW113 (*psaE*_H87A_), pEW114 (*psaE*_H141A_), pEW115 (*psaE*_H153A_), and pEW116 (*psaE*_H155A_). These plasmids were conjugated into YPA18 (the Δ*psaEF* mutant), and integration of these plasmids into the chromosome was identified by selection on BHI plates with Kan_50_ and Irg_2_.

### *gfp* transcriptional reporter assay.

To analyze *psaA* promoter activity, a *gfp* transcriptional reporter plasmid (pEW102) was electroporated into the indicated strains and assayed as previously described (18). Saturated cultures grown in unbuffered BHI were subcultured to an OD_600_ of 0.2 in buffered BHI and grown for 8 h with aeration at 37°C. Relative fluorescent units (RFU) were measured using a Synergy HT microplate reader (BioTek Instruments, Winooski, VT) and normalized to OD_600_. Data are represented as RFU/OD_600_ ± standard deviation.

### PsaF stability assay.

Saturated cultures of YP6 (WT) were subcultured to an OD_600_ of 0.2 in BHI buffered to pH 6.3 and grown for 8 h at 37°C. Cells (OD = 10) were pelleted, washed once with PBS adjusted to pH 6.3, split into equal volumes, pelleted, and suspended in PBS adjusted to either pH 6.3 or 7.3, and incubated at 37°C for 4 h. At 0 h, 2 h, and 4 h, cells (OD = 2) from each condition were collected and prepared for Western blot analysis.

### Western blot analysis.

Saturated cultures grown in unbuffered BHI were subcultured to an OD_600_ of 0.2 in BHI buffered to the indicated pH and grown for 8 h at 37°C. PsaE, PsaF, and PsaA were analyzed as previously described (18). Briefly, cell lysates were resuspended in Laemmli buffer, boiled, separated via SDS-PAGE, and transferred to a polyvinylidene difluoride (PVDF) membrane. Loading of protein samples was qualitatively assessed via Ponceau S staining. Anti-PsaE, anti-PsaF, and anti-PsaA sera were used as previously described (18). Anti-IgG horseradish peroxidase (HRP)-conjugated secondary antibodies were used at a titer of 1:20,000.

### Statistical analysis and reproducibility.

Analyses were performed using Prism 8 (GraphPad Software, San Diego, CA). All experiments were performed at least three times, with biological triplicates in each assay. Unless otherwise noted, representative assays are shown.
