# Supplementary material for: PsaF Is a Membrane-Localized pH Sensor That Regulates psaA Expression in Yersinia pestis
Source: J Bacteriol. 2021 Jul 22;203(16):e00165-21. doi: 10.1128/JB.00165-21 (PMC8407435; doi:10.1128/JB.00165-21)
Supplement: Supplemental file 1 — Fig. S1 and S2. Download JB00165-21_Supp_1_seq14.pdf, PDF file, 1.1 MB [file jb00165-21_supp_1_seq14.pdf]

Supplemental Data

PsaF is a membrane-localized pH sensor that regulates *psaA* expression in *Y. pestis*

Joshua D. Quinn, Eric H. Weening, Virginia L. Miller

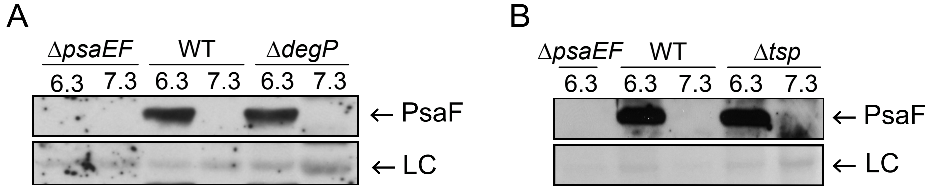

Fig S1. Levels of PsaF remain low at pH 7.3 in Δ*degP* and Δ*tsp* mutants. In-frame deletions of *degP* and *tsp* were constructed to generate the Δ*degP* (YPA425) and Δ*tsp* (YPA350) mutants, respectively, and these strains in addition to WT (YP6) and the Δ*psaEF* mutant (YPA18) were grown at 37°C in BHI buffered to pH 6.3 and pH 7.3 and whole cell lysates were prepared and used to analyze PsaF via western blot.
(LC; loading control)

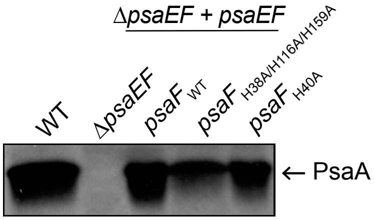

Fig S2. The three histidine residues unique to PsaF are not essential for the production of PsaA. Native and mutant *psaF* alleles were introduced into the Δ*psaEF* mutant (YPA18) to generate derivatives of the Δ*psaEF* mutant expressing wild-type *psaF* (YPA260), *psaF*$_{H38A/H116A/H159A}$ (YPA273) or *psaF*$_{H40A}$ (YPA271) and these strains in addition to WT (YP6) and the Δ*psaEF* mutant (YPA18) were grown at 37°C in BHI buffered to pH 6.3 and whole cell lysates were prepared and used to analyze PsaA via Western blot.
